# Nanomedicine Strategies for Management of Drug Resistance in Lung Cancer

**DOI:** 10.3390/ijms23031853

**Published:** 2022-02-06

**Authors:** Mohamed Haider, Amr Elsherbeny, Valeria Pittalà, Valeria Consoli, Maha Ali Alghamdi, Zahid Hussain, Ghalia Khoder, Khaled Greish

**Affiliations:** 1 Department of Pharmaceutics and Pharmaceutical Technology, College of Pharmacy, University of Sharjah, Sharjah 27272, United Arab Emirates; zhussain@sharjah.ac.ae (Z.H.); gkhoder@sharjah.ac.ae (G.K.); 2 Division of Molecular Therapeutics and Formulation, School of Pharmacy, University of Nottingham, Nottingham NG7 2RD, UK; amr.elsherbeny@nottingham.ac.uk; 3 Department of Drug and Health Science, University of Catania, 95125 Catania, Italy; vpittala@unict.it (V.P.); valeria_consoli@yahoo.it (V.C.); 4 Department of Biotechnology, College of Science, Taif University, Taif 21974, Saudi Arabia; mahaasg@agu.edu.bh; 5 Department of Molecular Medicine, Princess Al-Jawhara Centre for Molecular Medicine, School of Medicine and Medical Sciences, Arabian Gulf University, Manama 329, Bahrain; khaledfg@agu.edu.bh

**Keywords:** nanomedicine, drug resistance, lung cancer, chemotherapeutic agents, drug delivery

## Abstract

Lung cancer (LC) is one of the leading causes of cancer occurrence and mortality worldwide. Treatment of patients with advanced and metastatic LC presents a significant challenge, as malignant cells use different mechanisms to resist chemotherapy. Drug resistance (DR) is a complex process that occurs due to a variety of genetic and acquired factors. Identifying the mechanisms underlying DR in LC patients and possible therapeutic alternatives for more efficient therapy is a central goal of LC research. Advances in nanotechnology resulted in the development of targeted and multifunctional nanoscale drug constructs. The possible modulation of the components of nanomedicine, their surface functionalization, and the encapsulation of various active therapeutics provide promising tools to bypass crucial biological barriers. These attributes enhance the delivery of multiple therapeutic agents directly to the tumor microenvironment (TME), resulting in reversal of LC resistance to anticancer treatment. This review provides a broad framework for understanding the different molecular mechanisms of DR in lung cancer, presents novel nanomedicine therapeutics aimed at improving the efficacy of treatment of various forms of resistant LC; outlines current challenges in using nanotechnology for reversing DR; and discusses the future directions for the clinical application of nanomedicine in the management of LC resistance.

## 1. Introduction

Lung cancer (LC) is recognized as the second most diagnosed type of cancer and the leading cause of cancer-related deaths globally [1]. According to the World Health Organization, 2.21 million cases of LC were reported in 2020 worldwide [2]. LC is classified into the categories of small cell lung cancer (SCLC) and non-small cell lung cancer (NSCLC). The pace of treatment of SCLC is mainly faster than NSCLC because of the ability of the tumors to spread quickly. NSCLC is less aggressive, but it is generally identified at the advanced stages. Around 80% of LCs are NSCLC, and they can be further subdivided into adenocarcinoma, squamous cell carcinoma, bronchioalveolar carcinoma, and large cell carcinoma. The remaining 20% of LCs show proper ties of neuroendocrine differentiation [3]. The current approaches for management of LC involve surgical removal of non-metastatic tumors, radiation, and chemotherapy. Chemotherapeutic agents inhibit rapidly dividing cancer cells but also affect normal cells with high proliferation rates (e.g. bone marrow, hair follicles, etc.), resulting in undesirable side effects that may require dose reduction or discontinuance of the therapy. This unfavorable outcome is mostly due to poor and random delivery of those agents [4].

In addition, the therapeutic effectiveness of chemotherapeutic agents is limited due to the development of DR in cancer cells [5]. Cancer DR is the ability of the tumor cells to develop a certain mechanism to overcome and resist the cytotoxic or inhibitory effect of the chemotherapeutic agent and therefore reduce the effectiveness of chemotherapy [6]. Currently, the failure of chemotherapy due to DR accounts for 90% of clinical metastasis cases [6]. To overcome DR, chemotherapeutic agents need to be administered at larger doses with higher frequency, which in turn may result in increased toxicity and lower patient survival rate. Alternatively, a combination of two or more chemotherapeutic agents may be administered to achieve a synergistic effect and reduce the rate of DR [7]. This approach has improved the effectiveness of chemotherapy but has not yet eliminated the side effects associated with non-specific uptake by normal cells.

The need for an alternative strategy to deliver chemotherapeutic agents to be relatively more selective in targeting malignant cells and overcome drug resistance has been the main focus of recent studies. Over the last two decades, nanotechnology has played a major role in the delivery of medicinal agents to overcome the obstacles of conventional therapy [8,9]. A range of different types of nanocarriers (1 to 500 nm) has been developed for the delivery of drug molecules, nucleic acid, and diagnostic agents [10,11,12,13,14,15].

**Figure 1:**
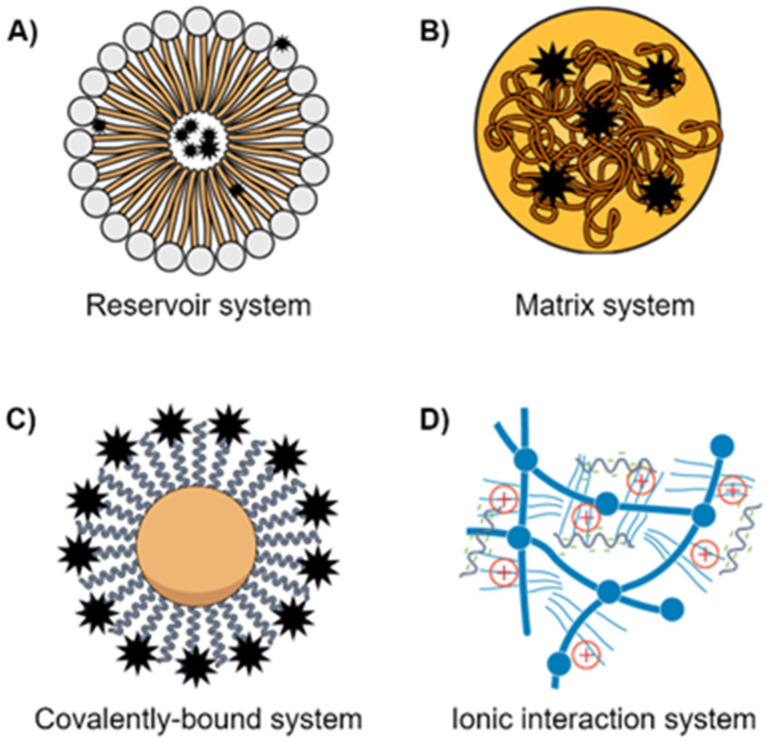
General therapy loading strategies in nanoparticle systems.

The use of nanoparticles (NPs) as carriers for chemotherapeutic agents has significantly improved their effectiveness, safety, stability, and pharmacokinetic profile [9,12,16]. Biocompatible nanocarriers can be tailored to suit the pathophysiology of the tumors and enhance the physicochemical properties of the drug and its permeability and retention time due to their unique sizes and possible surface modifications [12,14,16,17]. Furthermore, improvement in drug targeting by encapsulation in suitable NPs reduces the adverse effects associated with chemotherapy as normal cells are protected from the cytotoxic effect of the anticancer drugs [12,16,18]. Several strategies have been used for the encapsulation and loading of the therapeutic agent on the nano system. The selected techniques depend on the formulation procedure, carrier system, and physio-chemical properties of the pristine agent (Figure 1). Anticancer drugs may be loaded within the empty core of the NPs (Reservoir system), distributed evenly within the polymer matrix (Matrix system), conjugated covalently to the nanocarrier (Covalently bound system), or have an ionic interaction between oppositely charged ions (Ionic interaction system) [19].

In this review, we present the different molecular mechanisms of DR in LC and discuss the use of several types of nanocarriers to improve the chemotherapeutic outcome after treatment of drug resistant LC with anticancer drugs. We also outline the current challenges in using nanotechnology for reversing DR and discuss possible directions for future research.

## 2. Nanomedicine Applications in Management of Lung Cancer Drug Resistance

### 2.1. Tumor Microenvironment

**Figure 2:**
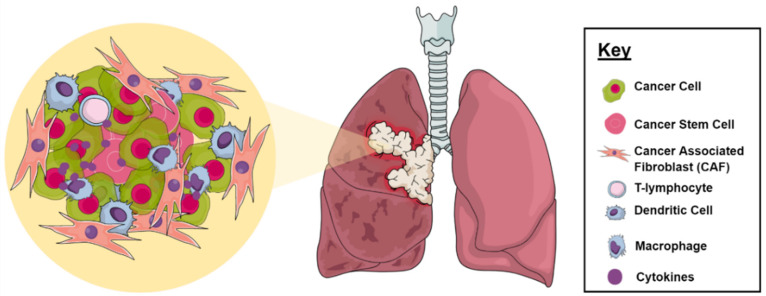
Representation of cellular components of TME in LC.

Cancer cells can control and influence the function of their environment by releasing complex signaling networks for their own benefits. Thus, cancer cells exist in a dynamic interaction with their surrounding environment that consists of cells and non-cell components, which allows them to evolve and grow, resulting in cancer progression, metastasis, and DR (Figure 2) [20]. TME includes stromal cells, which mainly consist of tumor epithelial cells, cancer-associated fibroblasts (CAFs), and immune cells. Non-cellular factors include extracellular matrix (ECM) components, such as growth factors, degradation enzymes, and inflammatory mediators. They also include exosomes and apoptotic bodies, in which they are known as extracellular vehicles (EVs). Moreover, the TME has special unique features, including hypoxia and an acidic environment [21,22,23]. These predominantly arise due to the insufficient blood supply and oxygen deprivation associated with the rapid and uncontrolled proliferation of cancer cells. Reactive oxygen species (ROS) are also generated well beyond their normal levels, which induces further mutations and carcinogenesis at the tumor site. Recent studies have outlined the ability of ROS to engage with CAFs in a two-way cross-talk, where CAFs increase the levels of ROS observed in the tumor tissue, promoting cancer growth and invasiveness, while ROS activate the CAFs through the upregulation of HIF1α [24]. The lung TME plays an imminent role in cancer cell resistance by interfering with the pharmacokinetic distribution of the anticancer agent [25]. The uncontrolled angiogenic activity, dense desmoplastic stromal layer, and abnormal interstitial and oncogenic pressures compromise the activity of the chemotherapeutics in inducing their cytotoxic activity [26]. In addition, the cross-talk between tumor and stromal cells modulates the response to these agents and reduces their potential within the microenvironment [27]. Poor immune cell infiltration and activation induced by the harsh conditions surrounding the tumor cells is of great importance to tumor proliferation and metastasis [28]. The infiltrating immune cells also play a dualistic role in the tumor tissue by either suppressing or promoting cancer progression according to their type and their effect on manipulating the unique setting of the tumor within the TME. This is highly dependent on the cross talk associated with the ongoing cytokines produced and their interaction with the tumor cells. Several different types of immune cells have been detected in the lung TME, including Natural Killer (NK) cells, T lymphocytes, macrophages, dendritic cells, Myeloid-derived suppressor cells, and B cells; however, their immunomodulatory properties within the tumor tissue have yet to be further explored [29]. These barriers lead to a decline in the intracellular accumulation of the anticancer agents, an increase in tumor-acquired resistance, and poorer clinical outcomes.

NPs have long been sought to overcome the limitations associated with the unique tumor setting within LC [25,30,31]. Given their inherent passive targeting enhanced permeation and retention (EPR) properties, alongside their ability to be actively formulated to utilize the internal factors regulating the TME, NPs may well improve the clinical response to the therapy in question [32]. Recent studies outlined how novel nano delivery systems could be prepared to exploit current TME hindrances in LC, such as the acidic nature of the microenvironment, increased accumulation of reactive oxygen species (ROS), expression of unique antigens at the tumor site, activation of immunogenic tissue as an immunomodulatory therapy, or external stimuli-triggered drug release, among others, achieving a relatively higher targeted activity in the tumor cell than with conventional therapy [33,34,35,36,37].

In a study by Yu-Lun Lo et al., a pH/redox-responsive micelle based on a poly(ε-caprolactone)-SS-poly (methacrylic acid) (PCL-SS-PMAA) diblock copolymer was fabricated for the dual drug delivery of paclitaxel (PTX) and cisplatin (CIS) [38]. The NPs were intended to utilize the acidic/ROS-rich TME for selective release of the chemotherapeutic agents while enhancing their intracellular accumulation. In vitro release studies revealed that the co-loaded formulation released almost 100% of its anticancer agents after 192 h when subjected to an acidic reducing environment (pH 5.5 + Dithiothreitol (DTT)) compared to only 40 % of the chemotherapeutic agents released at normal conditions (pH 7.4 with no DTT). This suggests that these NPs can maintain their integrity until exposed to the TME, incurring selective drug release. Cell apoptosis studies also revealed that treatment of NCI-H520 LC cells with the dual loaded PTX/CIS cross-linked micelles resulted in a 1.77-fold increase in cell death compared to the free drugs [38]. Similarly, redox-responsive manganese dioxide NPs (MnO2 NPs) stabilized with biocompatible polymers polyvinylpyrrolidone (PVP) and polyacrylic acid (PAA) were synthesized and analyzed using magnetic resonance (MR) imaging and measurement of cytotoxic activity on gefitinib-resistant LC cell lines. MnO2 NPs showed glutathione (GSH)-responsive dissolution and subsequent enhancement in MR imaging. In addition, the NPs induced a significant selective cytotoxic effect on NSCLC cells upon X-ray irradiation without a noticeable damage. Therapeutic efficacy was also achieved when the cells were treated with MnO2 NPs in hypoxic conditions [39]. It would be quite interesting to see these particles applied on 3D-co cultures, organoids, or in vivo and the results compared to current therapeutic regimens to mimic, to a greater extent, the potential selectivity of these particles.

In a different study, PTX was loaded in the N-succinyl-palmitoyl-chitosan acid responsive micelles decorated with the cRGDyK peptide to inhibit NSCLC. The cRGDyK peptide is an integrin ligand analogue that can target tumors via specific binding integrin receptors overexpressed in the tumor neo-vasculature but poorly expressed in resting endothelial cells and most normal organs. The authors suggested that these particles were acid responsive since the drug release at pH 5.3 was almost 20% higher than at pH 7.4 (65% and 45%, respectively, after 72 h). While this statement is true to an extent, it lacks accuracy since results were comparable at both pH settings. A more comprehensive approach could have been achieved had the authors compared the time required for all the encapsuled drugs to be released from the nanocarriers at both pH conditions. Nevertheless, when looking at the cytotoxicity of the functionalized micelle on A549 human NSCLC, the IC50 was 5x and 4x lower when compared to Taxol formulation and the unfunctionalized formula, respectively. This suggests that the cRGDyK peptide may well increase cellular uptake into the tumor tissue. The authors further supported their claim by studying the tumor uptake properties on tumor-bearing mice after 24 h. The ex vivo images of excised organs revealed that the accumulation of cRGDyK functionalized micelles in the lungs was more remarkable than that of unfunctionalized micelles. In vivo anti-tumor activity showed a significantly lower tumor mass in mice treated with PTX/cRGDyK-SPCS micelles than those treated with Taxol and PTX/SPCS micelles, thereby indicating that the functionalized drug-loaded micelles possessed the strongest inhibitory efficiency on the tumor [40].

Enhancing the immunotherapeutic outcome of current immunotherapy using NPs in LC has also been explored through the preparation of a combinatorial chemotherapy/immunotherapy Polo-like kinase 1 (PLK1) inhibitor (Volasertib)—loaded mesoporous silica NPs decorated with an PD-L1 antibody. PLK1 is an important mitotic kinase that is overexpressed in LC-promoting oncogenesis and tumor metastasis. PD-L1 expression in tumor cells inhibits tumor-directed cytotoxic CD8+ T cell activity by binding to the PD-1 receptor in T cells and suppressing their function. Therefore, a combinatorial delivery system is for the co-delivery of the PLK1 inhibitor. The PD-L1 antibody is thought to selectively kill tumor cells while upregulating PD-L1 expression in surviving cancer cells and/or increasing the density of tumor-infiltrating lymphocytes, providing an opportunity to achieve a targeted therapeutic activity in a positive feedback manner. The NPs-based immunotherapy showed a significant reduction in the effective doses of volasertib and the PD-L1 antibody by five-fold in a metastatic lung in vivo tumor model by actively mediating CD8+ T cells, allowing the immune cells to induce their cytotoxic activity on the cancer cells. These results clearly demonstrate the influence of targeting the TME on improving the clinical outcomes of current therapy [41]. Accordingly, exploiting the molecular pathways and interactions that govern the TME could potentially enhance the current approach to therapy using a novel nano drug delivery systems (DDS).

### 2.2. Multidrug Resistance

**Figure 3:**
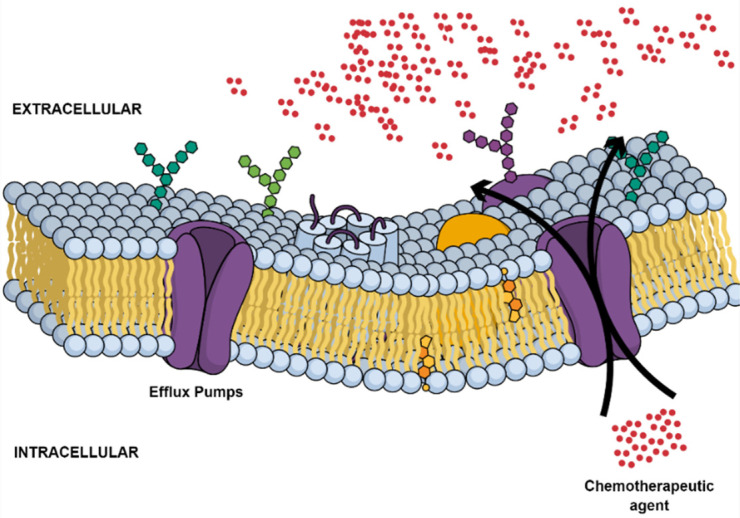
Drug efflux from the intracellular compartment of cancer cells.

Tumor multidrug resistance (MDR) remains a major obstacle that continues to hinder the effective progression of current curative cancer therapy in LC [42]. Innate and acquired phenotypes have been frequently identified as major cancer cell defense mechanisms following exposure to chemotherapeutic regimens [43]. Until now, several MDR mechanisms have been increasingly linked to members of the ATP-binding cassette (ABC) membrane pumps with 48 identified genes [44]. A number of these efflux transporters, including P-glycoprotein (P-gp; ABCB1; MDR1), breast cancer resistance protein (BCRP; ABCG2), and MDR-associated protein 1 (MRP1; ABCC1), have been recognized as reducing the efficacy of anticancer agents in tumor cells through a noticeable decrease in their intracellular accumulation in an ATP-dependent manner (Figure 3) [45]. Commonly used chemotherapeutic agents, including taxanes, platinum compounds, and gemcitabine, fall victim to these pathways [46,47,48,49].

Other non-ABC drug transporters in LC, such as lung resistance protein (LRP), have shown their ability in reducing the biodistribution of anticancer agents in the tumor microenvironment, reducing the overall efficacy of the medication regimen [44,50]. In a study that examined the mortality rate of patients expressing MDR and LRP transporters in NSCLC, survival rates were greatly diminished upon overexpression of these efflux transporters when compared to pump-free tumors, showing their imminent role in the chemoresistance of the disease [51]. Drug transporter-independent mechanisms also play a prominent role in the development of MDR in LC. An important superfamily of antiapoptotic proteins known as the B-cell lymphoma (Bcl-2) appear to be upregulated in LC, promoting cytotoxic resistance through the dysregulation of apoptosis in tumor cells [52,53,54]. In addition, mutations in the p53 transcriptional factors that regulate the expressions of numerous genetic materials have brought about considerable MDR in LC [55,56,57]. Thus, there is a substantial need for the development of novel systems to overcome the MDR shortcomings of current chemotherapy.

Recent studies have explored the potential role of nano DDS in overcoming several MDR processes in in vitro and in vivo studies on LC. It was suggested that anticancer drug-loaded NPs can evade efflux transporters due to the ability of NPs to enter the cells in large amounts by endocytosis rather than diffusion, then release the drug inside the cell at a perinuclear site away from the efflux pumps [58]. Another strategy involves using nanocarriers for codelivery of the P-gp inhibitor and the chemotherapeutic agent. In a study by Liang Xu et al., doxorubicin (DOX) was co-loaded with cyclosporin as a P-gp inhibitor in a poly (lactic-co-glycolic acid) PLGA-based nano vector and applied to a PTX-resistant A549 cell line (A549-TAXOL). The co-loaded formulation showed a significant decrease in cellular viability after 72 h relative to free DOX and DOX-loaded NPs. These results suggest the significance of P-gp inhibition on achieving chemo sensitization and improving cellular cytotoxicity outcomes. In vivo studies on A549-TAXOL xenografts subcutaneously injected into female BALB/c nude mice also revealed a significantly lower tumor volume with the co-loaded formulation relative to the free drug [59,60].

Similarly, when the photosensitizer 5,10,15,20-Tetrakis(4-hydroxy-phenyl)-21H,23H-porphine (pTHPP) was loaded into a PLGA-lipid hybrid NPs, the IC50 of the NPs was 523x and 45.2x lower than etoposide and PTX, respectively, on etoposide-resistant A549 cell lines (A549RT-eto). Treatment with free pTHPP in the presence or absence of light irradiation did not produce any cytotoxic effects in both A549 and A549RT-eto cells due to its very poor solubility and inability to be uptaken into the cells. The hybrid NPs enhanced the solubility of the free agent and achieved a higher cytotoxic effect when compared to conventional chemotherapy. Similar results were also obtained when the optimum NPs were used on detachment-induced MDR acquired by A549 cells cultured as floating cells under non-adherent conditions mimicking metastasizing cancer cells in the blood/lymphatic circulation. While these results are very promising, having cells cultured in a 2D monolayer may lead to an overestimation of the potential of these particles. Further studies on 3D co-culture models and/or in vivo assays may more closely resemble clinical outcomes [61].

In a different study, functionalized PTX-liposomes with d-α-tocopheryl polyethylene glycol 1000 succinate-triphenylphosphine conjugate (TPGS1000-TPP) as a mitochondrial targeting molecule and P-gp inhibitor were prepared and tested on drug-resistant human LC A549/cDDP cells. The targeted liposomal particles had a size of 80 nm and an encapsulation efficiency of >85%. They demonstrated an almost 40% increase in cytotoxicity compared to the free chemotherapeutic agent. In addition, the % apoptosis rate was 4% higher in resistant cell lines relative to PTX alone. Further studies revealed that the targeting PTX liposomes significantly enhanced the cellular uptake, initiating a cascade of caspase 9 and 3 reactions, thereby activating the pro-apoptotic Bax and Bid proteins and suppressing the anti-apoptotic Bcl-2 protein, which then enhanced the apoptosis by acting on the mitochondrial signaling pathways. In vivo studies on A549/cDDP xenografts subcutaneously injected into Female BALB/c nude mice revealed a 37% increase in the tumor volume inhibitory rate in the targeting PTX liposomes over the free drug, suggesting the ability of the DDS to overcome MDR pathways. However, it is important to note that the drug-free formulation showed a 30% cytotoxicity effect, which raises the concern of whether these particles could inherently be cytotoxic on normal cells, which requires further investigation [62].

The use of NPs to overcome other MDR pathways in LC by encapsulating genetic materials has also been explored. It was suggested that NPs inhibit the expression and function of efflux transporters by targeting microRNAs (miRNA), which are special non-coding RNAs that play an important role in protein expression and cellular transfection [63]. Shutting Ma et al. co-loaded survivin siRNA and a tetravalent platinum complex of cisplatin (Pt (IV)) prodrug into a protamine/hyaluronic acid nanocarrier coated with polyglutamic acid (PGA) for the treatment of platinum-resistant LC. Survivin is a cancer biomarker and a member of the anti-apoptosis family that has been found to be overly expressed in drug-resistant tumor cells. Silencing this pathway using siRNA was investigated in this study to observe its relative efficacy in overcoming CIS transporter-independent MDR pathways. Survivin siRNA was loaded into an NPs to overcome its poor biodistribution in vivo and achieve increased intracellular accumulation. Cytotoxicity results in 2D A549/cDDP cell lines after 24 h revealed a slight increase in % apoptosis in the co-loaded formulation relative to CIS. This unexpected poor outcome drove the researcher to further investigation since the drug release from the NP formulation was achieved after 191 h (97.3% for pH 5.0, 29.7% for pH 6.5). Western blot analysis showed that the amount of survivin protein expression of NP-siRNA/Pt (IV) was higher than that of the survivin siRNA due to the incomplete in-vitro release of survivin siRNA from the NP after 24 h. In vivo experiments performed on A549/DDP tumor-bearing nude mice showed that treatment with the co-encapsulated formulation resulted in highest tumor inhibition rates (82.46%) compared to free CIS (62.52%) after 14 days of treatment. Therefore, despite the poor 2D cellular outcomes, in vivo models demonstrated higher efficacy, with the dual loaded formulation highlighting the importance of in vivo models in exploring the potential of these formulations. However, it would have been interesting to also compare these results to free siRNA in vitro and in vivo to visualize the importance of loading such entities in NPs over the free agents [64].

Self-assembled polyjuglanin NPs loaded with DOX and anti-Kras siRNA were also formulated for attenuating MDR in human LC. Down-regulation of the Kras gene by siRNA has previously caused defective abilities of proliferation, clonal formation, migration, and invasion of cancer cells. In vitro studies were carried out using DOX-resistant A549/DOX and CIS-resistant H69/CIS human LC cell lines to investigate the effects of the NPs on MDR. The results showed that the combination therapy achieved a higher in vitro cytotoxicity result in both cell lines by oncogene dose-dependently reducing Kras, P-gp, and c-Myc, expression while improving p53 genetic modulation in drug-resistant cells. These results were further supported by in vivo studies where the NPs formulation achieved reduced tumor growth compared to the free DOX, accompanied with reduced KI-67 and enhanced TUNEL-positive levels in drug-resistant xenografted BALB/c nude mice [65]. In this, NPs clearly demonstrate their versatility in loading diverse agents and achieve MDR reversal by overcoming multiple molecular pathways in LC, showing their potential in enhancing the therapeutic actions of current and future regimens.

### 2.3. Cancer Stem Cells

Currently, it is well accepted that a subpopulation of LC cells residing within the tumor tissue exhibits unique biological phenotypes and characteristics with stem cell capacities, including lineage differentiation and self-renewal [66]. In addition, they can further undergo invasion, metastasis, tumorigenesis, chemoresistance, and tumor relapse and can escape immune surveillance. These cells will be henceforth referred to as cancer stem cells (CSCs) [67]. Studies have shown the vital role CSCs play in the occurrence and development of LC tissue, outlining their significance in mediating all cancer hallmarks. Recent studies suggest that the “stemness” of tumor cells may be caused by genetic mutations to specific genes, including TP53, or acquired through the activity of cancer microenvironment substances, such as interleukins, nitric oxide, or hypoxic conditions. This situates the CSCs within a setting rich with external signals, such as cytokines, growth factors, extracellular matrices, and other physicochemical factors and surrounded by a variety of cells, such as immune cells, stromal cells, endothelial cells, and perivascular cells [66,67,68,69,70]. Several biomarkers have already been identified within LC, including ALDH1, CD133, CD44, CD166, CD20, and others [68]. Understanding the CSC environment may well provide effective therapeutic strategies to overcome the aforementioned barriers, thereby achieving an improved therapeutic outcome and a lower rate of tumor recurrence.

The use of NPs to selectively target the overexpressed biomarkers and improve the therapeutic activity of cytotoxic agents on CSCs has been explored. Hyaluronic acid functionalized/all-trans-retinoic acid- (ATRA) loaded albumin-based cationic NPs were prepared and evaluated in CD44 overexpressed CSCs in in vivo lung metastasized tumor models. Pharmacokinetic biodistributions revealed a selective uptake of the HA-decorated NPs in the tumor tissue of the mouse, with a significant reduction in tumor growth relative to the pristine drug [71]. Similarly, Dandan Liu et al. formulated a heat shock protein inhibitor-loaded silica-coated Fe3O4 magnetic NP decorated with anti-CD20 CSCs-specific antibodies to kill both cancer cells and CSCs. The multifunctional thermoresponsive/immunomodulant/chemotherapeutic NPs demonstrated an almost 98% eradication of human lung CSCs within 30 min of external application of an alternating magnetic field (AMF). Further in vivo studies revealed that the combinatorial therapy significantly suppressed tumor growth and metastasis in lung CSC xenograft-bearing mice, demonstrating a relatively high efficacy while maintaining good biocompatibility and targeting capability [72]. This illustrated that such NPs could effectively serve as a platform for further exploration on selective antitumor activity on normal cancer cells and CSCs alike.

Epithelial-mesenchymal transition (EMT) is another crucial process involved in promoting cancer invasion and metastasis [73]. Cancer cells undergoing EMT show similar characteristics to CSCs, such as increased chemoresistance and oncogenic activity while entering a period of dormancy upon EMT completion. Therefore, it can be safely concluded that EMT could be among the contributing factors in inducing and generating the CSCs and their niche within the TME [74]. Accordingly, interfering with the EMT pathway is likely to halt the process of CSC development and promote effective therapeutic outcomes. In a study by Chiou et al., miR145 was loaded into polyurethane-short branch-polyethylenimine (PU-PEI) NPs and delivered into lung adenocarcinoma CSCs (LAC-CSCs). Tumor growth and metastasis appeared to be reduced upon delivery of the loaded carrier systems in in vivo studies to xenograft tumors. In addition, xenografts appeared to be to more sensitive to chemoradiotherapies, prolonging the survival times of tumor-bearing mice [75]. Another important regulator of signaling pathways associated with CSCs is the SOX2 gene [76]. Andey et al. prepared a cationic lipoplex for the targeted delivery of the SOX2 small interfering RNA (CL-siSOX2) to SOX2-enriched, CSC-derived orthotopic, and xenograft lung tumors in CB-17 SCID mice. The authors presented well-established work that demonstrates the significance of CL-siSOX2 in inhibiting the expression of stemness markers in xenograft tumors, including SOX2, NANOG, c-MYC, and KLF4. These lipoplexes also reduced tumor volume in mice, suggesting that such effects were attributed to the crucial role of SOX2 in the regulation of signaling pathways associated with CSCs [77]. The results of these studies show the potential of NPs as effective carriers for a range of therapeutic modalities, improving their relative selectivity and overcoming the limitations associated with CSCs in the lung tissue.

### 2.4. Metabolic Inactivation of the Anticancer Drugs

**Figure 4:**
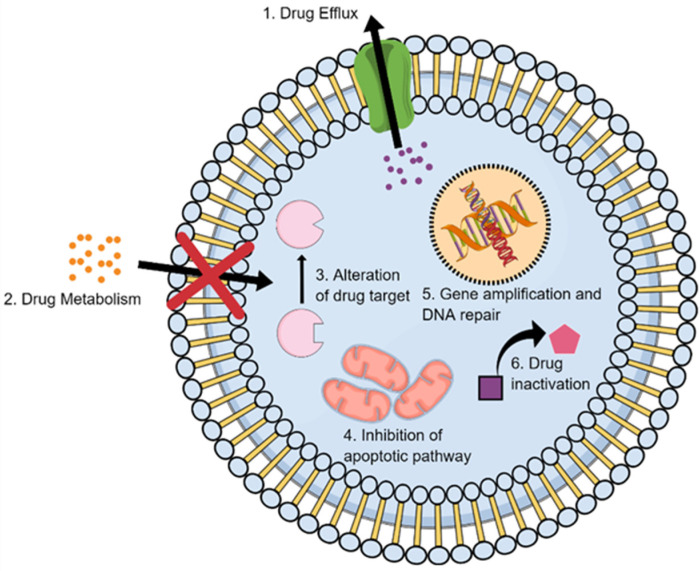
Different mechanisms of DR in LC.

Drug detoxification is considered a key resistance mechanism in several types of malignant tumors (Figure 4). Each population of cancer cells can respond differently to anticancer drugs due to the associated genomic variation [78]. The metabolism of chemotherapeutic agents can progress intracellularly and/or extracellularly, eventually affecting the overall efficacy of the given anticancer agent [79].

The metabolism of anticancer drugs in the body involves two phases. In the initial phase, cytochrome-P450 (CYP-P450) enzymes may act on the functional groups of the anticancer drugs and modify them by oxidation, reduction, or hydrolysis reactions. Examples of functional groups include hydroxyl (-OH), amino (-NH2), and carboxylic acid (-COOH). The second phase involves further modification of those altered functional groups by glucuronidation, sulfonation, and conjugation of the amino acid, creating more hydrophilic and polar drug metabolites that are readily excreted [80]. Metabolism may also involve methylation and acetylation reactions, which could terminate the activity of the drugs. These conjugation reactions normally occur in specialized cells in the liver to protect the host from the toxicity of anticancer drugs [81,82,83]. However, similar pathways have been demonstrated at the tumor site in many types of cancer, including LC [83]. Augmented metabolic activation in cancer cells can eventually influence the pharmacokinetics and/or pharmacodynamics of anticancer drugs [84,85].

For anticancer prodrugs, such as cyclophosphamide, the concentration of the active drug molecules at the tumor site is dependent on the metabolism of the prodrug, and therefore, the metabolic activity can be a limiting factor for the effectiveness of the treatment. The overproduction of specific enzymes at tumor sites can be used to render a prodrug into cytotoxic metabolites strictly at those sites [86]. Many studies have exploited this phenomenon in delivering nano-based chemotherapeutics, such as using HPMA enzyme-linked systems that are specifically cleaved by tumor-specific enzymes, such as cathepsin B [87].

Furthermore, there are many examples of ongoing research utilizing key metabolic pathways in overcoming anticancer DR. For instance, the glutathione detoxification pathway is a system used by the cells to maintain an intrinsic homeostatic state. It plays an essential role in detoxification of the peroxides that are generated from oxygen radicals as a result of treatment with anticancer drugs [88]. It consists of glutathione (L-g-glutamyl-l-cysteinyl-glycine, GSH), related enzymes, and glutathione S-conjugate complex export protein (GS-X pump). The glutathione can form a conjugate with xenobiotics, drugs, and the sulfhydryl groups of several proteins, which are then catalyzed by the enzyme glutathione S-transferases (GSTs) and effluxed from the cells via the adenosine triphosphate-dependent GS-X pump [88,89]. The significance of this system in the anticancer resistance varies according to the type of cancer cells and the difference in the levels of enzymes related to the detoxification process. In some cases, an increased level of the enzyme is involved in the synthesis of glutathione γ glutamyl cysteine synthetase (γ GCS) and the GSTs conjugation enzyme, which are the key functional enzymes for resistance. The GST family, also known as MAPEG proteins, is a large superfamily found in cytosolic, mitochondrial, and microsomal cancers [90,91]. Theses enzymes can increase the cancer cells’ resistance directly through the detoxification of anticancer drugs or indirectly via mitogen-activated protein kinase pathway (MAPK) inhibition within the RAS/MAPK signaling pathway in the cells. This was reported to be present in higher levels in lung tumors than in the normal bronchioles and alveoli [17,18,92,93].

Platinum-based compounds, such as CIS, are commonly used in the treatment of patients with advanced stages of LC. When the drug reaches the cancer cells it causes an increased level of ROS and DNA damage, leading to cell apoptosis [94,95,96]. The resistance to LC congruently develops by inactivating the platinum drugs via many resistance mechanisms, including increase in the DNA repair, reduction in cellular uptake, and anti-apoptosis. Several studies on the glutathione metabolic system showed that the active SH-group in glutathione can bind to the platinum-based drugs and negate their DNA targeting due to their high affinity with the anticancer drugs. With the elevated level of glutathione in the cells, the drug efflux by the GS-X pump is further enhanced, and the cells become CIS-resistant [97,98,99].

Cancer cells display a high level of protein expression of GST and γ GCS often seen in the CIS-resistant lung, as shown in several studies. This increase may be explained as an attempt to detoxify the drug. However, the internalized drugs that are conjugated to GSH can inhibit the MAPK kinase pathway, which works on killing tumor cells via activation of the JNK/MAPK pathway, leading to apoptosis. Therefore, this pathway is inhibited in the resistant LC cells by increased GST expression [97]. In addition to the overexpression of GSH, cancer cells develop resistance to chemotherapy through the overproduction of superoxide dismutase SOD or/and sulfur-containing macromolecules, such as metallothioneins (MTs). MTs were found in high levels in the resistant NSCLC with squamous cell lung carcinoma and adenocarcinoma. They are intracellular proteins with high amounts of cysteine that act as precursors for GSH synthesis [100,101,102]. The concentration of GSH in the extracellular fluids of the resistance cancer cells is 100–1000 times higher than in the sensitive cancer cells. In this aspect, such significant differences can be useful in drug delivery to resistant LC cells, as the drug release can be enhanced through GSH-dependent thiolysis [103,104]. Accordingly, several studies reported the synthesis of nanocarriers with reducible linkers, such as thioether bonds [104], disulfide bonds [105,106], and di-selenide bonds [107], that release the drug in the presence of GSH. The most common and simplest bond addressed in this regard is the disulfide bond that can be inserted into polymeric micelles nano delivery systems as part of a linker between two blocks of polymers. Upon internalization of the polymeric micelles by resistant cancer cells with high levels of GSH, the linker disulfide bond is cleaved, leading to disassembly of the micelles and release of the drug [108,109,110,111,112,113,114,115]. Wang et al. synthesized polymeric micelles composed of PEG, polyethyleneimine (PEI) blocks, and ATP-depleting Pluronic P123 bound together with a disulfide bond and loaded with PTX- and siRNA-targeting polo-like kinase1 (PLK1) that acts in downregulation of ATP and interferes with the cancer cell metabolism. The results showed an increase in PTX and siRNA release, accompanied by a reduction in drug efflux by fast ATP-depletion of the resistant cancer cells [116]. Similar results were obtained for NPs loaded with thiolated anticancer drugs. For example, dendrimer-encapsulated gold NPs loaded with thiolated anticancer drugs showed the same GSH-dependent enhanced drug release [117].

### 2.5. Inhibition of the Cell Death

Among the many mechanisms put in place by cancerous cells in chemoresistance is indeed the evasion of apoptosis program (Figure 4). Apoptosis, also known as type I cell death, is a regulated cell death (RCD) characterized by alterations in cell morphology, shrinkage of cytoplasm, plasma membrane blebbing, and chromatin condensation, which result in the formation of small vesicles, known as apoptotic bodies [118,119]. It is well known that dysregulated apoptosis in cancer cells promotes resistance to anticancer drugs [120,121].

Apoptosis consists of two distinct pathways: intrinsic and extrinsic. Stress stimuli, such as DNA damage, initiate the intrinsic apoptotic pathway and trigger mitochondrial outer membrane permeabilization (MOMP), which eventually promotes the activation of caspase-9. In turn, caspase-9 activates the caspases-3, -6, and -7 responsible for apoptosis execution. On the other hand, the extrinsic apoptotic process starts with the binding of specific ligands to cell surface death receptors, activating caspase-8 and subsequently the other executioner caspases [121]. Cancer cells escape the apoptosis program, exploiting several mechanisms, which not only results in primary tumor progression and metastasis but also abrogation of therapeutic response to chemotherapy [122].

Gene mutations have been observed to be one of the many factors implicated in apoptosis evasion by cancer cells. This involves the generation of abnormal transcription products, leading to a loss or gain of function for several proteins, dysregulation of cellular homeostasis, and resistance to apoptosis. Examples of gain function mutations are represented by the catalytic subunit of PI3K (PI3KCA) mutations (E542K, E545K and H1047K) that cause sustained PI3K pathway activation. Instead, loss function mutations can occur at the expense of BAX, p53, and Phosphatase Tensin Homolog (PTEN) genes [123,124,125,126].

The modulation of extrinsic and intrinsic apoptotic signals effects can be reconducted to a family of structurally distinct inhibitor of apoptosis proteins (IAPs): cellular (cIAP1, cIAP2), surviving, X-linked (XIAP), neuronal (NIAP), livin, BIR-ubiquitin conjugating enzyme (BRUCE), and testis specific (Ts-IAP). IAPs expression is specifically upregulated during diseases progression and DR onset, hence the interest towards IAPs as potential targets for resistant cancer treatment [127,128]. For instance, X-linked IAP (XIAP) is upregulated in LC cells and enhances apoptosis inhibition.

Many studies have focused on finding the possible correlation between the usefulness of Bcl2 family proteins and chemotherapy outcomes to make plausible predictions. A correlation between Bcl2 expression and response to chemotherapy was established in patients with LC [129,130]. However, none of the investigated apoptosis-related proteins (Bcl2, Bax, Bcl-xl, Bag1, FAS, FASL) could be helpful in predicting the response to drug treatment for breast cancer [131]. Interestingly, this observation was also confirmed in other studies involving vinorelbine- and docetaxel-combined treatment in patients with NSCLC [132]. Thus, there is a rising conflict in literature, which, to date, does not allow scientists to establish any direct connection between disrupted apoptotic pathways and chemotherapy failure.

Several approaches have been explored to date on the use of pro-apoptotic NPs as potential chemoresistance therapy in human LC. For instance, a novel pro-apoptotic drug–drug conjugate was obtained by Shim and co-workers through the conjugation of the pro-apoptotic peptide drug (SMAC; Ala-Val-Pro-Ile-Ala-Gln, AVPIAQ) and cathepsin B-cleavable peptide (Phe-Arg-Arg-Gly, FRRG) to DOX, resulting in SMAC-FRRG-DOX that self-assembled into NPs. Upon cellular uptake, the NPs were cleaved to obtain pro-apoptotic SMAC and cytotoxic DOX specifically in cancer cells that overexpress cathepsin B, inducing a synergic effect of the combined molecules in a metastatic LC model [133].

Wang et al. designed and synthesized a TPP-Pluronic F127-hyaluronic acid (HA) (TPH), with a mitochondria-targeting triphenylphosphine (TPP) head by formation of an ester bond. PTX-loaded TPH (TPH/PTX) nanomicelles showed great physical properties and efficacy in A549-resistant cells. TPH/PTX initiated MOMP through inhibition of antiapoptotic Bcl-2, leading to the activation of caspase-3 and caspase-9. This study was able to demonstrate the actual perks of targeting mitochondria of cancer cells to counteract and prevent DR, as well as the ability of nanomicelles to enhance mitochondrial-specific delivery [134,135,136].

Furthermore, gene therapy involving re-establishing pro-apoptotic response using NPs-based technologies for delivery has been the focus of many studies. The transfection of the p53 gene by cationic solid lipid NPs (SLN) and PLGA in LC cells has been reported. The cationic SLNs were prepared by the melt homogenization method and then formulated by mixing tricaprin (TC) as a core, 3beta [N-(N′,N′-dimethylaminoethane) carbamoyl] cholesterol (DC-Chol), dioleoylphosphatidylethanolamine (DOPE), and Tween 80 in various ratios. Treatment exhibited an efficient re-establishment of wild-type p53 function, restoring the apoptotic program in NSCLC [137,138].

### 2.6. Alteration of Drug Targets

Resistance to chemotherapeutic agents can be due to alteration in their targets at the tumor sites. These changes occur due to molecular modifications that may begin by mutation in DNA and alterations in protein expression, resulting in a decrease in the affinity of the drugs with their binding targets and DR (Figure 4). For example, treatment of SCLC with DOX in combination with platinum drugs inhibits the topoisomerase enzymes in the cells by intercalation between the DNA bases, causing inhibition to the enzyme gyrase that is responsible unwinding the structure of DNA during the DNA replication and ultimately causing DNA breakage. Many of resistant cancer cells can survive this treatment by modifying topoisomerase II gene expression and hence altering the target of DOX [78,139].

A similar DR mechanism was also reported for anticancer drugs that target specific signaling kinases, such as the epidermal growth factor receptor (EGFR) family [26,140,141]. In this case, a mutation commonly occurs in the receptor kinase, leading to over-activation of these kinases and their downstream signaling molecules such as Ras, Src, and MEK. Many of these kinases become constitutively active and promote uncontrollable cell growth. In some cancers, if the drug targets molecules of the signaling pathways, the resistant cancer cells tend to activate alternative molecules. The mutations in the EGFR in anaplastic lymphoma kinase (ALK) fusion gene-positive LC after the patient was treated with crizotinib serve as an example. Acquired resistance to the drug occurred via (ALK)-mutations, such as EGFR (L1196M and C1156Y), and some patients had other mechanisms of resistance with both mutations and increase in ALK gene copy number [142,143,144]. The single-nucleotide mutations, such as L1196 and G1269A, were reported in some cases to cause crizotinib resistance in NSCLC [145]. However, sometimes, the same effect of the mutation that causes over-activation can be found via gene overexpression. Overexpression of certain receptors in some LCs with a mutation in the EGFR tyrosine kinase domain causes drug-acquired resistance that may occur after the long-term use of drugs inhibitors targeting this kinase [145]. EGFR-targeted liposomal nanoparticles (EGFR-LP) were developed for the treatment of NSCLC resistance to drugs as erlotinib and afatinib, determined by mutations in the tyrosine kinase (TK) domain of EGFR [146]. Ramanathan and colleagues have re-ported a novel DNA-based colorimetric assay for the detection of early EGFR mutation using unmodified gold nanoparticles (GNPs) [147].

The resistance to EGFR therapy could also involve alteration of the PTEN-PI3K-AKT pathway. The PTEN refers to the phosphatase and tensin homolog, which is a tumor suppressor gene that impedes tumor growth via the inhibition of the Akt oncogene that promotes cell survival by inactivating of some apoptosis mediators [148]. The loss of PTEN results in cancer cells resistance for EGFR inhibitors, such as erlotinib, that initiates negative regulation in the PI3K-AKT pathway leading to PI3K activation and tumor progression [149,150].

Angiogenesis inhibitors, such as bevacizumab, can face resistance from cancer cells that involves alteration of VEGF tyrosine kinase receptors (VEGFR) and binding with Neuropilin-1 (NP1) and/or Neuropilin-2 (NP2) [151,152]. When bevacizumab blocks VEGF-A, NP1, and NP2, resistant cancer cells use alternate VEGFR-1 and VEGFR-2 pathways, leading to angiogenesis and tumor progression. In NSCLC, the elevated level of expression for both NP1 and NP2 in tissues was found to be correlated with tumor growth [153].

Co-delivery is a targeting strategy applied mainly in molecular-targeting therapy for the treatment of NSCLC. The first approved epidermal growth factor receptor (EGFR) drug is gefitinib a tyrosine kinase inhibitor (TKI) that is used for the treatment of EGFR mutation in NSCLC. The long-term treatments for patients with gefitinib can result in the development of DR. Approximately 90% of EGFR mutations found deletions in exon 19 and single missense and secondary mutation in exon 20 in 50% of patients with the secondary T790M mutations (EGRPT790M) associated with the resistance to gefitinib [140,154,155,156,157,158,159,160,161]. The resistance to gefitinib occurs due to mutated methionine (M) residue, which blocks the interaction between the anticancer drug and the active EGFR pocket. In this case, a co-delivery system can be used to overcome resistance issues. Peng et al., used mannose-modified liposomal and HER-2 antibodies as a co-delivery system (tLGV) to treat NSCLC with EGRPT790M-mutation [162]. Another liposomal co-delivery system involved the use of the PD-L1 nanobody as a ligand in gefitinib-loaded liposomes for treatment of NSCLC with EGFRT790M-positive mutation [163]. Overall, nano-systems could benefit from combating this resistance mechanism through enhancement of the initial drug dose to the tumor tissues through passive targeting. In addition, nanoformulations could enhance the TKIs formulations bioavailability and enhance their peak plasma level through protection from metabolizing enzymes, as discussed in Section 2.4.

### 2.7. Enhancing DNA Repair

DNA repair involves a tangled network of repair mechanisms dictated by the specific kind of stimuli and damage to which cells are exposed (Figure 4). These mechanisms include mismatch repair (MMR), nucleotide excision repair (NER), base excision repair (BER), direct reversal (MGMT, ABH2, ABH3), homologous recombination (HR) and nonhomologous end joining (NHEJ) pathways. For instance, ionizing radiation induces double-strand breaks (DSBs) mainly repaired by nonhomologous end joining (NHEJ) pathways. On the other hand, mono- and bifunctional alkylators can induce DNA-base modifications interfering with DNA synthesis, which can be reversed in a mismatched repair-dependent manner [44,164,165].

Inhibition of DNA repair systems may be a potential strategy to sensitize cancer cells to chemotherapeutic drugs and increase their efficacy. However, even if disrupting DNA repair systems may block the resistance to chemotherapeutic agents, it can also be responsible for the development of new mutations due to genomic instability [166].

CIS-resistant cancer cells showed higher levels of DNA damage repair. In addition, it was noted that inhibition of NER pathways can significantly enhance tumor cells’ sensitivity to CIS. The enhanced DNA repair capability in lung-CSCs was associated with an extensive activation of DNA repair genes in response to CIS treatment, suggesting it may be the main mechanism involved in resistance insurgence [167,168]. Studies have also highlighted an inverse correlation of ERCC1 (NER pathways) with response to platinum therapy in LC [169]. Apurinic/apyrimidinic endonuclease 1 (APE1) is considered a crucial BER pathway protein due to its activity as intermediate in the processing of potentially cytotoxic DNA damage sites. Moreover, APE1 seems to have a dual role, depending on its cellular localization, where it carries out DNA repair in the nucleus. However, in the cytoplasm, its primary role is assumed to be the regulation of mitochondrial DNA repair, possibly together with the regulation of various transcription factors. In LC cells, APE1 is often overexpressed, especially in CIS-resistant cancers [170,171].

Ongoing studies have proved the actual potential of targeting DNA repair elements to prevent or overcome DR. Coadministration of natural compounds, such as curcumin (CUR), enhanced CIS apoptotic activity on CIS-resistant lung adenocarcinoma cells through the inhibition of FANCD2 mono-ubiquitination and inactivation of the Fanconi anemia (FA)/BRCA pathway, which is a DNA cross-link damage repair pathway responsible for cellular resistance regulation towards DNA cross-link agents [172]. A similar effect was also reported by Hong et al. in their work using CIS prodrug (CDDP) and CUR co-encapsulated NPs in the treatment of NSCLC. The results of the study showed that the co-delivery of both chemotherapeutic agents using PLGA-based NPs induced a synergistic response, increased the therapeutic efficacy, and overcame DR [173].

In a different study, polypeptide-based nanocarriers were used for combined targeting of DNA repair and DNA damage-induced cell cycle checkpoint pathways through the inhibition of both mitogen-activated protein kinase, (MAPK)-activated protein kinase 2 (MAPKAPK-2 or MK2), and Xeroderma Pigmentosa Group A (XPA) [174]. This treatment strategy could be further enhanced by adding CIS to reduce CIS resistance and improve therapeutic outcomes.

Another successful approach was based on loading demethoxycurcumin (DMC) into a self-assembled amphiphilic carbomethyl-hexanoyl chitosan (CHC) nanomatrix. The drug-loaded nanomatrix significantly reduced CIS-induced DR through the suppression of excision repair cross-complementary 1 (ERCC1) in NSCLC. Moreover, the bioavailability and targeting capacity toward cancer cells were improved by preparation of a DMC-polyvinylpyrrolidone core phase, followed by the encapsulation in a CHC shell to form a DMC-loaded core-shell hydrogel NPs (DMC-CHC NPs) [175].

### 2.8. Gene Amplification

DR due to gene amplification is estimated to occur in 10% of the cancers. It involves an increase in the number of copies of certain oncogenes inside the resistant cancer cells to several hundred times more than the drug-sensitive cancer cells. This eventually lead to the production of related oncoproteins in large amounts per cell (Figure 4). For instance, the MET gene amplification is found to affect 5–20% of EGFR-TKI-treated NSCLC patients who develop resistance to TKI drugs. HER2 amplification also has been recognized as a rare resistant mechanism in lung adenocarcinoma occurring in 1–2% of total cases in patients and tends to be up to 13% in NSCLC patients with resistance to EGFR-TKIs [176,177,178,179]. The MET is a proto-oncogene that encodes itself into MET proteins (c-MET), which can result in an increase in tyrosine kinase signaling and excessive cellular division [180]. There is a link between the MET and the third-generation EGFR-TKIs resistance in the EGFR mutant (EGFRm) NSCLC cell line (HCC827/ER). Acquired resistance to erlotinib due to the amplified MET gene in the cells and associated with hyperactivated MET protein also leads to resistance to both osimertinib and rociletinib [181]. The use of a small-molecule MET inhibitor or genetic knockdown to the expression of MET successfully increased the sensitivity of HCC827/ER cells to osimertinib and effectively inhibited the cell growth in vitro and in vivo [181,182].

The amplification of genes was also detected in the MDR1/ABCB1 chromosomal region that encodes the P-gp (P-gp/ABCB1) with overexpression of the ATP-binding cassette pumps in resistant LC cells after being treated with PTX. This resulted in a decrease in cellular accumulation of PTX, an increase in its efflux out of the cancer cells, and the development of resistance to the drug [47,183,184,185]. The encapsulation of chemotherapeutic agents into NPs or their conjugation to polymeric carriers allow them to evade the ABC drug efflux pumps as they become unrecognizable as substrates to be exported. In one study, anti-MRP-1 and anti-Bcl2 siRNA were encapsulated in combination with DOX in liposomes. The DDS targeted both pump and non-pump mediated cellular LC resistance, leading to suppression of efflux pumps and an increase in drug accumulation inside resistant LC cells [186].

### 2.9. Epigenetic Alteration Caused Drug Resistance

Although all cells of the human body have the same exact genes, epigenetic alterations regulate the way genome can be read. These are changes in the chemical structure of DNA that do not change the nucleotide coding sequence but have a profound effect on gene expression. Epigenetic alterations may occur due to the adding of and exposure to environmental factors, such as diet, exercise, drugs, and chemicals [187,188,189]. Methylation and acetylation of DNA are two well-studied epigenetic events that significantly alter the expression of genes, resulting in the upregulation of oncogenes and/or downregulation of tumor suppressor genes and development of cancer DR [190].

In eukaryotes, histones mainly serve as a structure guide for several enzymes to provide the necessary platform for RNA polymerase access to its target. Histone acetyltransferases (HATs) and histone deacetylases (HDACs) are essential enzymes that regulate histone acetylation, which is the pivotal focus of several studies on post-translational modification mechanisms. Most of the common features displayed by cancerous cells, such as the evasion of apoptosis, increased angiogenesis, and metastasis progression can be linked to epigenetic modulation and to HDAC. A number of studies highlighted the multiple roles of HDAC, suggesting it as a potential target for chemotherapy and establishing the basis for the development and use of HDAC inhibitors (HDACi) as co-adjuvant for many anticancer agents for treatment of NSCLC [191,192]. PTX co-administration with HDACi SNOH-3 showed reversed DR in PTX-resistant NSCLC cells characterized by overexpression of HDAC1 [193]. Sharma et al. demonstrated the ability of a subset of stem-like cells in NSCLC cell lines to undergo chromatin remodeling following treatment with erlotinib and CIS, which allow the development of drug insensitivity [194]. However, despite the myriad of pre-clinical work supporting HDACi efficacy as adjuvant of chemotherapy in treatment of NSCLC, they have demonstrated modest efficacy as single agents in clinical trials.

The use of nanocarriers for the delivery of epigenetic agents has noticeably enhanced their ability as co-adjuvants to re-sensitize cancer cells after the onset of anticancer DR. Studies on using HDACi-loaded NPs in combination with chemotherapy and radiotherapy demonstrated the enhancement of anti-proliferative effects [195]. For example, to improve the bioavailability of the histone deacetylase inhibitor vorinostat (VOR) and its efficacy in the treatment of multidrug resistant cancers, solid lipid NPs (SLNs) were used as carriers. Treatment of resistant LC cell line with VOR-SLNs resulted in improved efficacy, elevated payload capacity, and a sustained release profile. The results also showed that lower doses of VOR-SLNs were required to obtain the same cytotoxic effect as free-VOR [196].

Other studies suggested that gefitinib resistance in patients with NSCLC may be correlated to EGFRT790M secondary mutation in those patients after treatment with gefitinib. Peng et al. reported the preparation of a dual-targeted liposome system for the delivery of vorinostat and gefitinib that is decorated with the anti-HER-2 antibody and mannose for targeting HER-2-overexpressing tumor cells and mannose receptor-expressed tumor-associated macrophages (TAMs), respectively. The drug-loaded immunoliposomes were able to reverse EGFRT790M-positive NSCLC resistance to gefitinib through the regulation of ROS/NOX3/MsrA axis and reconfiguration of TAMs [162,197]. Examples of preclinical studies on nanomedicine targeting epigenetic alteration and other mechanisms of DR in LC are mentioned in Table 1.

### 2.10. Clinical Studies Using Nanotechnology for Management of DR in LC

The potential use of NPs in the treatment of drug-resistant LC was explored in many clinical trials (Table 2). In 2012, the FDA approved the first nano-formulation for treatment of NSCLC patients, Abraxane, which consists of solvent-free albumin-bound PTX-NPs based on its significant improved clinical trial outcomes [212]. Other nano-formulations have been the subject of various clinical trials and showed promising therapeutic outcomes in the treatment of resistant LC (http://www.clinicaltrials.gov). Examples of clinical studies that aimed to evaluate the efficacy and safety of NPs loaded with various therapeutic agents to target LC at different stages are discussed in Table 2.

## 3. Current Limitations and Future Perspectives of Nanomedicine Aimed at Overcoming Drug Resistance

Cancer therapy is the primary and most attractive field for nanomedicine applications, also thanks to the history of success of Doxil and Abraxane as the main representatives mentioned in several clinical studies [46,47]. Nanoscale DDSs hold promise for new insights and innovative solutions to overcome conventional chemotherapy issues, allowing precise delivery of anticancer agents to specific malignant sites and ensuring efficient cellular internalization. This can lead to complete tumor eradication and can be potentially useful to overcoming chemo-resistance in cancers [48].

Even though many NPs have achieved important milestones as potential therapies [49,50,51], most of them still fail to meet the clinical standards. In addition, clinically approved NPs have proved to be effective in reducing drugs toxicity; yet their application has not always resulted in a better clinical outcome. Deficiency in understanding of the biological mechanisms, complex design, and the absence of accurate characterization techniques in addition to the high cost of manufacturing have jeopardized nanomedicines’ clinical translation.

## 4. Biological Aspects

Biodistribution modulation, biological barrier breaching, and complex heterogeneity of human diseases are the main biological factors that must be taken in consideration to design and produce NPs able to reach clinical trials. Random distribution and accumulation at nontarget sites remain the central obstacles for the development of effective nanomedicines. Therefore, an alternative development strategy that relies on a disease-driven approach rather than the conventional formulation-driven approach is currently needed. This means that DDS engineering should be the core of research. To do so, a solid understanding of the connections between biology and technology must be attained [213]. Studying the biological processes that control barriers’ functions and their involvement in disease progression, together with discovering new materials, will ensure the development of NPs capable of overcoming the obstacles for efficacious and site-specific delivery [17].

## 5. Formulation Drawbacks

In order to deal with the complexity of malignant tumors, it is crucial to have a consistent and highly reproducible formulation prior to the clinical phases [214]. Novel antineoplastic delivery systems are more and more based on the development of multifunctional NPs with specific targeting and image contrast-enhancing properties added to the basic structure of the carriers. The addition of specific tags to the nanocarriers adds further complexities to the synthesis process, increases the costs, introduces complex interactions and effects in vivo, and hinders regulatory affairs. Targeted therapeutics are surely attractive and most of the time seem to be the right solution to the conventional chemotherapeutic issues. Nevertheless, their synthesis and purification, together with choosing the most fitting and effective ligand-receptor couple, can make their realization challenging and risky, not always resulting in a positive outcome or a feasible production. Moreover, it is known that even the smallest modification in the structure can affect the binding features, leading to steric hindrances, conformational changes and less efficacy. The complexity further increases when it involves nanomedicines carrying more than just one active compound, as this can significantly affect their pharmacological profile. In order to overcome these limitations, novel bioconjugation methods are in developing phases. Among them, the click chemistry concept has been regaining a great deal of interest, basing new NPs design on easier drug production in order to guarantee straightforward and economic synthesis of large libraries of new compounds and to reduce the costs [215,216,217]. Moreover, to obtain clinical approval, it is fundamental for the entire process to rely on a stable and reproducible product. Unfortunately, in most of the cases, NPs tested in preclinical studies are tendentially synthesized in small batches, and their scale-up for higher production is not always possible, even for clinical studies [218,219]. There is also the need for specific regulatory guidelines and a streamlined approval process, which address the complexity of nanomedicine characterization, together with pharmacological and toxicological issues [220]. All these elements represent the biggest challenges that prevent nanomedicines from reaching clinical phases.

## 6. New Approaches for the Use of Nanomedicine in the Treatment of Resistant LC

Novel receptor-specific targeting strategies exploiting the peculiar characteristics of the tumor microenvironment could be useful in overcoming several complications and hurdles associated with targeted NPs and could significantly reduce cancer resistance to chemotherapy. While conventional approaches rely on environmental stimuli to guide NPs delivery and localization, some new formulations aim to manipulate their path externally. For instance, it was observed that iron-based NPs loaded with anticancer drugs can be directly guided by a magnetic field gradient in the precise tumor location [221]. Alexiou et al. have been using magnetic NPs, in particular the commercially available ferrofluids, as drug carriers injected intravenously. The application of an external magnetic field at the tumor site post-injection resulted in accumulation of the NPs at the tumor area and reduced the systemic toxicity of the drug. Furthermore, magnetic NPs may function as carriers for multiple anticancer agents, e.g., genes, cancer-specific antibodies, and radio nuclides [221].

Alternatively, photodynamic therapy (PDT) has emerged in the last decade as a potential therapeutic approach for cancer management. Since most photosensitizers have predominantly hydrophobic characteristics, appropriate delivery systems are required [222,223,224,225,226]. In vitro studies on LC cell lines using nanocarriers loaded with photosensitizers showed interesting results that involved the induction of mitochondrial dysfunction through the decoration of targeting moieties on the nanocarriers. The interesting work of Shi et al. focused on liposomal-based nanomedicine (L@BP) loaded with a mitochondria-anchored photosensitizer (Cy-Br) and PTX and showed successful release and accumulation of both agents in the tumor site. In addition, it showed an enhanced therapeutic efficacy on PTX-resistant LC cells, setting the basis for its potential use on MDR cancers [227].

Another emerging strategy to prevent DR involves using NPs for the delivery of siRNAs. Delivering different siRNAs could concurrently silence several genes, including those genes responsible for DR. The siRNAs’ physicochemical features impede their cellular uptake since they are not capable of easily crossing phospholipid membranes. Therefore, appropriate carriers and development of new RNAi technology are required. Genome-editing technology may provide a platform for the development of newer and better approaches.

One of the possible strategies that can be useful in developing more effective NPs DDS for management of resistance to anticancer drugs is the integration of biocompatible compounds. For example, Reshma et al. have proposed the use of biopolymers, such as tamarind seed polysaccharide PST, to prepare PTX-loaded NPs through epichlorohydrin crosslinking. PST-PTX NPs were able to downregulate multidrug resistance related proteins, as P-gp and BCRP, in resistant cells, suggesting the potential of these particles as MDR inhibitors [228].

## 7. Conclusions

There are many mechanisms involved in the development of DR to chemotherapy in treatment of LC. Nanotechnology has shown promising results in the delivery of chemotherapeutic agents through increases in their circulation time, offers of precise multiple targeting, enhancement of drug accumulation at the tumor site, improvement in cellular uptake into the cytoplasm and/or nuclei of cancer cells, and effective carrying of combinations of therapeutic payloads. Nanotechnology has also shown great potential in overcoming DR in LC by inhibiting some mechanisms, such as the overexpression of drug efflux transporters, tumor microenvironments, activation of DNA repair pathways, prevention of cell apoptosis, and cancer stem cells. Currently, various nanomedicines have been widely used, and some others are already in clinical trials. It is therefore expected that the current progress of NPs development may provide new strategies for the treatment of cancer resistance. Although some successes have been achieved in the nanomedicine preclinical applications, many challenges must be overcome to speed up the clinical transformation of nanomedicine.

## Figures and Tables

**Table 1:** Preclinical studies nano-based strategy to overcome DR in LC.

Type of Nanomedicine	Drug Load	Targets	Cells/Cancer	References
PCL-SS-PMAA micelles	CIS/PTX	pH/redox responsive	In vitro NCI-H358 LC cells	[38]
Magnetic NPs	Manganese dioxide	Redox responsive/radiosensitive	In vitro hypoxic-induced gefitinib-resistant PC9 human LC cells (PC9GR)	[39]
cRGDyK-SPCS micelles	PTX	pH responsive/protein targeting	In vitro Luc-A549 LC cells and in vivo Luc-A549 cells implanted subcutaneously into the right upper flanks of a female nude mouse	[40]
Mesoporous silica nanoparticle decorated with PD-L1 antibody (ARAC)	Volasertib	Immune cells modulation	In vivo LLC-JSP murine LC cells (200K) inoculated in right flank of C57BL/6 female mice	[41]
PLGA NPs	DOX/Cyclosporin	P-gp	In vitro PTX-resistant LC A549 cell line (A549-Taxol) and in vivo A549-Taxol cells implanted subcutaneously into female BALB/c mice	[60]
TPGS1000-TPP	Paclitaxel	P-gp and mitochondrial targeting	In vitro CIS-resistant human LC cells, A549/cDDP cells and in vivo A549/cDDP xenografts subcutaneously injected into female BALB/c nude	[62]
Graphene	TRAIL + DOX	FADD	Human LC	[198]
Gold	Gefitinib	EGFR	LC (in vitro)	[199]
Gold	Erlotinib	EGFR	Human adenocarcinoma and NSCLC (in vitro)	[200]
Liposomal	Erlotinib DOX	EGFR	Human breast and LC	[201]
Liposomal	siRNA (MRP1/BCL2) DOX	MRP1/BCL2	Human LC	[187]
Liposomal	Lonidamine + epirubicin (in a separate liposomal formulation)	Mitochondrial hexokinase	Human LC	[202]
Liposomal	TRAIL + DOX (in separate NPs)	FADD	Human LC	[203]
Nanoliposomes in combination with radiation therapy	CIS (CDDP), radiation therapy	CIS alkylating and crosslinking DNA, sensation to radiation lesions	Human Lewis lung carcinoma A549 cells subcutaneously inoculated into C57BL/6N mice, n ivivo model	[204]
LCP NPs	siRNA (c-Myc) Gemcitabine monophosphate	c-Myc	Human LC	[205]
LCP NPs	SiRNA (VEGF) Gemcitabine monophosphate	VEGF	Human LC	[206]
(MPEG-PCL) micelles	CUR + DOX	ABC pumps/NF-κB	Murine LC	[207]
Polymeric micelles	Paclitaxel and survivin shRNA, which down-regulate survivin gene expression by RNA interference	Co-delivery of drug and gene-enhanced antitumor effect	LC	[208]
PLGA NPs	Cyclosporin A + DOX	P-gp	Human LC	[60]
PEG-PLA NPs	Gefitinib, cyclosporin A	EGFR	LC	[209]
PEG 1000 succinate-containing micellar NPs	PTX, fluorouracil (5-FU)	Inhibition of P-gp, inhibition of cell division by PTX, irreversible inhibition of thymidylate synthase, synergism of PTX/5-FU	H460/TaxR human NSCLC overexpressing P-gp in vitro mode	[210]
SHR-A1403 Polymeric NPs	Anti-c-Met monoclonal antibody (c-Met mAb) conjugated to a micro-tubule inhibitor	c-Met	Non-small cell LC cells	[211]
PCL-SS-PMAA: Poly(ε-caprolactone)-SS-poly(methacrylic acid), cRGDyK-SPCS: micelles N-succinyl-palmitoyl-chitosan decorated with cRGDyK peptide, TPGS1000-TPP: liposomes decorated with d-α-tocopheryl polyethylene glycol 1000 succinate-triphenylphosphine, PI3K: phosphoinositide 3-kinase, EGFR: epidermal growth factor receptor, VEGFR: vascular endothelial growth factor receptor, BCL2: B-cell lymphoma 2, LCP: lipid/calcium/phosphate, MPEG-PCL: methoxy poly(ethylene glycol)-poly(caprolactone), PEG-PLA: polyethylene glycol-block-poly(D, L-lactic acid), MDR1: multidrug resistance 1, MRP1: multidrug resistance-associated protein, FADD: Fas-associated protein with death domain, TRAIL: tumor necrosis factor-related apoptosis-inducing ligand, BCL2: B-cell lymphoma 2; siRNA: small interferin RNA				

**Table 2:** Examples of clinical trials using nanocarrier-based DDS in treatment of LC. *

Type of NPs	Cargo/Therapy	Status	Patient/Ccondition	Stage	ClinicalTrials.gov Identifier:
Liposomes	Drug: LY01610 (Irinotecan hydrochloride liposome injection)	Recruiting	SCLC	Phase 2	NCT04381910
Liposomes	Device: Liposomal DOX combined with ifosfamide	Unknown	SCLC	Phase 2	NCT01872416
Liposomes	Drug: PLM60	Recruiting	SCLC	Phase 2	NCT04352413
Liposomes	Drug: MRX34	Terminated	SCLC	Phase 1	NCT01829971
Polymeric-PEG	Drug: ADI-PEG 20 (Arginine deiminase pegylated)	Terminated	SCLC	Phase 2	NCT01266018
Polymeric-PEG	Drug: LCL161 Drug: Topotecan Drug: Pegylated GCSF (PEG-GCSF)	Terminated	LC	Phase 1, Phase 2	NCT02649673
Polymeric-PEG	Drug: Pegylated irinotecan	Completed	SCLC	Phase 2	NCT01876446
Polymeric-PEG	Drug: Pegylated irinotecan	Recurrent Small Cell	LC	Phase 2	NCT01876446
Polymeric-PEG	Drug: PEG-rhG-CSF	Unknown	SCLC	Not Applicable	NCT03776604
Polymeric-PEG	Drug: ADI-PEG 20	Completed	Solid tumors NSCLC	Phase 1	NCT01497925
Polymeric-PEG	Drug: Pegylated recombinant human endostatin (PEG-ENDO)	Recruiting	Solid tumors NSCLC	Phase 1	NCT04413227
Polymeric-PEG	Drug: PEG-rhG-CSF	Completed	Malignant Solid Tumor LC	Phase 4	NCT02805166
Polymeric-PEG	Drug: YPEG-rhG-CSF, 20 μg/kg, single s.c. at 48 h after chemotherapy for each experimental cycle Drug: YPEG-rhG-CSF, 30 μg/kg, single s.c. at 48 h after chemotherapy for each experimental cycle Drug: YPEG-rhG-CSF, 45 μg/kg, single s.c. at 48 h after chemotherapy for each experimental cycle Drug: PEG-rhG-CSF, 100 μg/kg, single s.c. at 48 h after chemotherapy for each experimental cycle	Completed		Phase 2	NCT02005458
Polymeric-PEG	Drug: ADI-PEG 20	Terminated	Non-squamous NSCLC	Phase 1	NCT02029690
NPs	Drug: EP0057 Drug: Olaparib	Recruiting	Lung neoplasms	Phase 1 Phase 2	NCT02769962
NPs	Drug: BIND-014	Completed	NSCLC	Phase 2	NCT01792479
NPs	Drug: BIND-014 (Docetaxel NPs for injectable suspension)	Completed	KRAS-positive patients with NSCLC Squamous cell NSCLC	Phase 2	NCT02283320
NPs	Drug: AGuIX Radiation: Radiotherapy	Recruiting	NSCLC	Phase 1, Phase 2	NCT04789486
Micelles	Drug: PTX (Genexol) Drug: PTX-loaded polymeric micelle (Genexol-PM)	Completed	NSCLC	Phase 2	NCT01023347
Micelles	Drug: PTX micelles for injection Drug: PTX injection Drug: CIS	Active, not recruiting	NSCLC	Phase 3	NCT02667743
Albumin	Drug: Nanoparticle albumin-bound PTX/carboplatin	Unknown	NSCLC	Phase 2	NCT01872403
Albumin	Drug: Carboplatin Drug: Erlotinib hydrochloride Drug: PTX albumin-stabilized nanoparticle formulation Radiation: Radiation therapy	Completed	LC	Phase 2	NCT00553462
Albumin	Drug: HLX10 Drug: Carboplatin and nab paclitaxel Drug: Placebo	Recruiting	NSCLC	Phase 3	NCT04033354
Albumin	Drug: Nanoparticle albumin-bound PTX	Unknown	NSCLC	Phase 2	NCT02016209
Albumin	Drug: Albumin paclitaxel Drug: Simvastatin	Recruiting	SCLC	Phase 2	NCT04698941
Albumin	Drug: PTX/Albumin-bound PTX Drug: IBI308	Recruiting	SCLC	Phase 2	NCT04056949
Radioactive 18F-Fluoropaclitaxel (FPAC)	Drug: FPAC	Terminated	LC	Phase 1	NCT01086696
NPs	Drug: TargomiRs	Completed	NSCLC	Phase 1	NCT02369198

* Source: https://clinicaltrials.gov/.
